# 1,3,6-Trimethyl­pyrano[4,3-*b*]pyrrol-4(1*H*)-one

**DOI:** 10.1107/S1600536809055627

**Published:** 2010-01-16

**Authors:** V. Krishnakumar, F. Nawaz Khan, Venkatesha R. Hathwar, P. Nithya, S. Suresh

**Affiliations:** a School of Advanced Sciences, VIT University, Vellore 632 014, Tamil Nadu, India; b Solid State and Structural Chemistry Unit, Indian Institute of Science, Bangalore 560012, Karnataka, India; c Department of Chemistry, Aarupadai Veedu Institute of Technology, Vinayaka Missions University, Paiyanoor, Chennai 603 104, Tamil Nadu, India

## Abstract

All the non-H atoms of the title compound, C_10_H_11_NO_2_, are almost coplanar [maximum deviation = 0.040 (3) Å]. The crystal structure is stabilized by C—H⋯O hydrogen bonds.

## Related literature

For general background to isocoumarins, see: Barry (1964 ▶). For related structures, see: Abid *et al.* (2006 ▶, 2008 ▶); Hathwar *et al.* (2007 ▶).
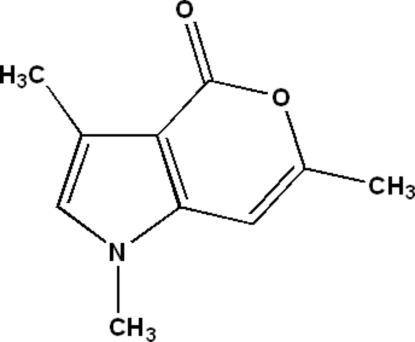


## Experimental

### 

#### Crystal data

C_10_H_11_NO_2_
*M* *_r_* = 177.20
Monoclinic, 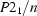

*a* = 7.5556 (7) Å
*b* = 8.4819 (8) Å
*c* = 14.3081 (14) Å
β = 93.870 (6)°
*V* = 914.86 (15) Å^3^
*Z* = 4
Mo *K*α radiation
μ = 0.09 mm^−1^
*T* = 295 K
0.33 × 0.28 × 0.15 mm

#### Data collection

Oxford Xcalibur Eos (Nova) CCD detector diffractometer
Absorption correction: multi-scan (*CrysAlis PRO RED*; Oxford Diffraction, 2009 ▶) *T*_min_ = 0.916, *T*_max_ = 0.987
7291 measured reflections
1692 independent reflections
1176 reflections with *I* > 2σ(*I*)
*R*_int_ = 0.034

#### Refinement

*R*[*F* ^2^ > 2σ(*F* ^2^)] = 0.051
*wR*(*F* ^2^) = 0.179
*S* = 1.18
1692 reflections
122 parameters
H-atom parameters constrained
Δρ_max_ = 0.27 e Å^−3^
Δρ_min_ = −0.18 e Å^−3^

### 

Data collection: *CrysAlis PRO CCD* (Oxford Diffraction, 2009 ▶); cell refinement: *CrysAlis PRO CCD*; data reduction: *CrysAlis PRO RED* (Oxford Diffraction, 2009 ▶); program(s) used to solve structure: *SHELXS97* (Sheldrick, 2008 ▶); program(s) used to refine structure: *SHELXL97* (Sheldrick, 2008 ▶); molecular graphics: *ORTEP-3* (Farrugia, 1997 ▶) and *CAMERON* (Watkin *et al.*, 1993 ▶); software used to prepare material for publication: *WinGX* (Farrugia, 1997 ▶).

## Supplementary Material

Crystal structure: contains datablocks global, I. DOI: 10.1107/S1600536809055627/bt5154sup1.cif

Structure factors: contains datablocks I. DOI: 10.1107/S1600536809055627/bt5154Isup2.hkl

Additional supplementary materials:  crystallographic information; 3D view; checkCIF report

## Figures and Tables

**Table 1:** Hydrogen-bond geometry (Å, °)

*D*—H⋯*A*	*D*—H	H⋯*A*	*D*⋯*A*	*D*—H⋯*A*
C10—H10*B*⋯O1^i^	0.96	2.46	3.404 (3)	170

Symmetry code: (i) 
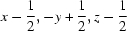
.

**Fig. 1.**
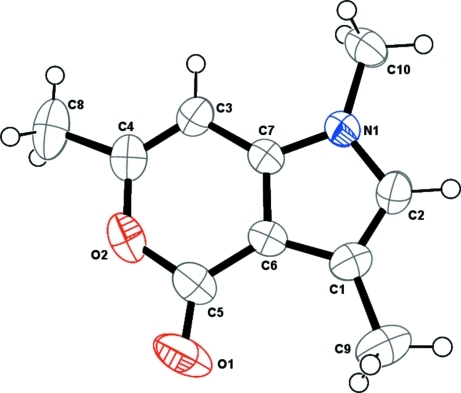
A view of the title complex, showing 50% probability displacement ellipsoids and the atom-numbering scheme.

**Fig. 2.**
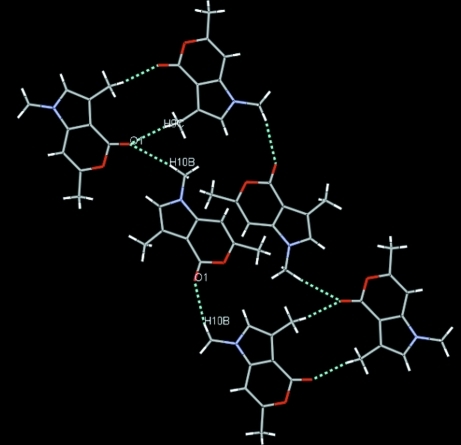
The packing diagram depicting C—H···O intermolecular interactions.

## supplementary crystallographic information

### Comment

Isocoumarins (Barry, 1964) are also useful intermediates in the synthesis of a variety of important compounds including some carbocyclic and heterocyclic compounds. In view of their natural occurrence, biological activities and utility as synthetic intermediates, we have synthesized the title compound, and reported herein its crystal structure.

### Experimental

A mixture of 2-(carboxymethyl)-1, 4-dimethyl-1*H*-pyrrole-3-carboxylic acid (2 mmol) and acetic anhydride (8 mmol) in the presence of pyridine was refluxed  for 4 h. Completion of the reaction was monitored by Thin Layer Chromatography. After completing of the reaction, the mixture was poured into crushed ice. The solids were separated and purified by silica gel column chromatography. The product was obtained with 90% yield.

### Refinement

All the H atoms   were positioned geometrically and refined using a riding model, fixing the bond lengths at 0.96 and 0.93 Å for CH_3_ aromatic CH, respectively. The displacement parameters of the H atoms were constrained as *U*_iso_(H) = 1.2*U*_eq_ (1.5*U*_eq_ for methyl) of the carrier atom.

### Crystal data

**Table:**

C_10_H_11_NO_2_	*F*(000) = 376
*M**_r_* = 177.20	*D*_x_ = 1.287 Mg m^−^^3^
Monoclinic, *P*2_1_/*n*	Mo *K*α radiation, λ = 0.71073 Å
Hall symbol: -P 2yn	Cell parameters from 1235 reflections
*a* = 7.5556 (7) Å	θ = 2.9–20.4°
*b* = 8.4819 (8) Å	µ = 0.09 mm^−^^1^
*c* = 14.3081 (14) Å	*T* = 295 K
β = 93.870 (6)°	Block, colorless
*V* = 914.86 (15) Å^3^	0.33 × 0.28 × 0.15 mm
*Z* = 4	

### Data collection

**Table:**

Oxford Xcalibur Eos (Nova) CCD detector diffractometer	1692 independent reflections
Radiation source: Enhance (Mo) X-ray Source	1176 reflections with *I* > 2σ(*I*)
graphite	*R*_int_ = 0.034
ω scans	θ_max_ = 25.5°, θ_min_ = 2.8°
Absorption correction: multi-scan (*CrysAlis PRO* RED; Oxford Diffraction, 2009)	*h* = −9→9
*T*_min_ = 0.916, *T*_max_ = 0.987	*k* = −9→10
7291 measured reflections	*l* = −17→17

### Refinement

**Table:**

Refinement on *F*^2^	Secondary atom site location: difference Fourier map
Least-squares matrix: full	Hydrogen site location: inferred from neighbouring sites
*R*[*F*^2^ > 2σ(*F*^2^)] = 0.051	H-atom parameters constrained
*wR*(*F*^2^) = 0.179	*w* = 1/[σ^2^(*F*_o_^2^) + (0.0888*P*)^2^ + 0.144*P*] where *P* = (*F*_o_^2^ + 2*F*_c_^2^)/3
*S* = 1.18	(Δ/σ)_max_ < 0.001
1692 reflections	Δρ_max_ = 0.27 e Å^−^^3^
122 parameters	Δρ_min_ = −0.18 e Å^−^^3^
0 restraints	Extinction correction: *SHELXL97* (Sheldrick, 2008), Fc^*^=kFc[1+0.001xFc^2^λ^3^/sin(2θ)]^-1/4^
Primary atom site location: structure-invariant direct methods	Extinction coefficient: 0.004 (1)

### Fractional atomic coordinates and isotropic or equivalent isotropic displacement parameters (Å^2^)

**Table:**

	*x*	*y*	*z*	*U*_iso_*/*U*_eq_	
N1	0.1293 (2)	0.4172 (2)	0.87406 (12)	0.0412 (5)	
O1	0.3720 (3)	0.1280 (3)	1.12304 (13)	0.0818 (7)	
O2	0.3792 (2)	0.3881 (2)	1.13262 (10)	0.0586 (6)	
C1	0.1778 (3)	0.1674 (3)	0.92158 (16)	0.0466 (6)	
C2	0.1079 (3)	0.2591 (3)	0.85100 (16)	0.0466 (6)	
H2	0.0534	0.2216	0.7951	0.056*	
C3	0.2612 (3)	0.5620 (3)	1.01528 (16)	0.0460 (6)	
H3	0.2372	0.6633	0.9929	0.055*	
C4	0.3421 (3)	0.5382 (3)	1.09968 (17)	0.0507 (7)	
C5	0.3331 (3)	0.2497 (3)	1.08310 (16)	0.0526 (7)	
C6	0.2454 (3)	0.2744 (3)	0.99253 (14)	0.0420 (6)	
C7	0.2128 (3)	0.4269 (2)	0.96087 (14)	0.0389 (6)	
C8	0.4000 (4)	0.6605 (4)	1.1693 (2)	0.0749 (9)	
H8A	0.3742	0.7631	1.1435	0.112*	
H8B	0.5253	0.6511	1.1842	0.112*	
H8C	0.3380	0.6465	1.2251	0.112*	
C9	0.1882 (4)	−0.0092 (3)	0.9237 (2)	0.0702 (9)	
H9A	0.1145	−0.0518	0.8725	0.105*	
H9B	0.1480	−0.0472	0.9818	0.105*	
H9C	0.3087	−0.0416	0.9181	0.105*	
C10	0.0743 (4)	0.5500 (3)	0.81496 (17)	0.0551 (7)	
H10A	0.0008	0.6192	0.8487	0.083*	
H10B	0.0085	0.5123	0.7597	0.083*	
H10C	0.1771	0.6063	0.7974	0.083*	

### Atomic displacement parameters (Å^2^)

**Table:**

	*U*^11^	*U*^22^	*U*^33^	*U*^12^	*U*^13^	*U*^23^
N1	0.0466 (11)	0.0379 (11)	0.0382 (10)	0.0025 (8)	−0.0049 (8)	0.0018 (8)
O1	0.1038 (17)	0.0781 (16)	0.0623 (12)	0.0317 (12)	−0.0034 (11)	0.0266 (11)
O2	0.0562 (11)	0.0777 (14)	0.0403 (9)	0.0065 (9)	−0.0082 (7)	−0.0015 (9)
C1	0.0506 (13)	0.0375 (13)	0.0521 (14)	−0.0005 (11)	0.0074 (11)	−0.0007 (11)
C2	0.0488 (13)	0.0463 (14)	0.0442 (12)	−0.0021 (11)	−0.0008 (10)	−0.0089 (12)
C3	0.0474 (13)	0.0415 (14)	0.0489 (13)	−0.0035 (10)	0.0016 (10)	−0.0050 (10)
C4	0.0443 (13)	0.0604 (17)	0.0471 (13)	−0.0021 (12)	0.0015 (10)	−0.0097 (11)
C5	0.0541 (15)	0.0589 (17)	0.0448 (13)	0.0102 (13)	0.0022 (11)	0.0066 (13)
C6	0.0432 (12)	0.0416 (13)	0.0409 (12)	0.0030 (10)	0.0017 (9)	0.0050 (10)
C7	0.0392 (12)	0.0400 (13)	0.0372 (11)	0.0023 (10)	0.0005 (9)	0.0033 (9)
C8	0.0654 (18)	0.096 (2)	0.0630 (17)	−0.0162 (17)	−0.0004 (13)	−0.0329 (16)
C9	0.085 (2)	0.0395 (16)	0.087 (2)	0.0000 (15)	0.0159 (16)	−0.0025 (15)
C10	0.0634 (16)	0.0556 (17)	0.0450 (13)	0.0061 (13)	−0.0055 (11)	0.0116 (11)

### Geometric parameters (Å, °)

**Table:**

N1—C7	1.357 (3)	C4—C8	1.483 (4)
N1—C2	1.388 (3)	C5—C6	1.431 (3)
N1—C10	1.452 (3)	C6—C7	1.387 (3)
O1—C5	1.206 (3)	C8—H8A	0.9600
O2—C4	1.380 (3)	C8—H8B	0.9600
O2—C5	1.403 (3)	C8—H8C	0.9600
C1—C2	1.353 (3)	C9—H9A	0.9600
C1—C6	1.430 (3)	C9—H9B	0.9600
C1—C9	1.500 (4)	C9—H9C	0.9600
C2—H2	0.9300	C10—H10A	0.9600
C3—C4	1.332 (3)	C10—H10B	0.9600
C3—C7	1.419 (3)	C10—H10C	0.9600
C3—H3	0.9300		
			
C7—N1—C2	108.41 (19)	N1—C7—C6	107.66 (19)
C7—N1—C10	125.64 (19)	N1—C7—C3	129.6 (2)
C2—N1—C10	125.94 (19)	C6—C7—C3	122.7 (2)
C4—O2—C5	124.21 (19)	C4—C8—H8A	109.5
C2—C1—C6	105.5 (2)	C4—C8—H8B	109.5
C2—C1—C9	127.3 (2)	H8A—C8—H8B	109.5
C6—C1—C9	127.2 (2)	C4—C8—H8C	109.5
C1—C2—N1	110.2 (2)	H8A—C8—H8C	109.5
C1—C2—H2	124.9	H8B—C8—H8C	109.5
N1—C2—H2	124.9	C1—C9—H9A	109.5
C4—C3—C7	117.4 (2)	C1—C9—H9B	109.5
C4—C3—H3	121.3	H9A—C9—H9B	109.5
C7—C3—H3	121.3	C1—C9—H9C	109.5
C3—C4—O2	121.3 (2)	H9A—C9—H9C	109.5
C3—C4—C8	126.8 (3)	H9B—C9—H9C	109.5
O2—C4—C8	111.9 (2)	N1—C10—H10A	109.5
O1—C5—O2	115.6 (2)	N1—C10—H10B	109.5
O1—C5—C6	129.6 (3)	H10A—C10—H10B	109.5
O2—C5—C6	114.8 (2)	N1—C10—H10C	109.5
C7—C6—C1	108.3 (2)	H10A—C10—H10C	109.5
C7—C6—C5	119.6 (2)	H10B—C10—H10C	109.5
C1—C6—C5	132.2 (2)		
			
C6—C1—C2—N1	0.3 (2)	O1—C5—C6—C7	179.4 (2)
C9—C1—C2—N1	−177.7 (2)	O2—C5—C6—C7	0.0 (3)
C7—N1—C2—C1	−0.5 (2)	O1—C5—C6—C1	−0.4 (4)
C10—N1—C2—C1	178.7 (2)	O2—C5—C6—C1	−179.8 (2)
C7—C3—C4—O2	0.6 (3)	C2—N1—C7—C6	0.4 (2)
C7—C3—C4—C8	−178.2 (2)	C10—N1—C7—C6	−178.74 (19)
C5—O2—C4—C3	−1.5 (3)	C2—N1—C7—C3	−178.8 (2)
C5—O2—C4—C8	177.5 (2)	C10—N1—C7—C3	2.1 (4)
C4—O2—C5—O1	−178.4 (2)	C1—C6—C7—N1	−0.2 (2)
C4—O2—C5—C6	1.1 (3)	C5—C6—C7—N1	179.96 (18)
C2—C1—C6—C7	−0.1 (2)	C1—C6—C7—C3	179.06 (19)
C9—C1—C6—C7	177.9 (2)	C5—C6—C7—C3	−0.8 (3)
C2—C1—C6—C5	179.7 (2)	C4—C3—C7—N1	179.6 (2)
C9—C1—C6—C5	−2.2 (4)	C4—C3—C7—C6	0.5 (3)

### Hydrogen-bond geometry (Å, °)

**Table:**

*D*—H···*A*	*D*—H	H···*A*	*D*···*A*	*D*—H···*A*
C10—H10B···O1^i^	0.96	2.46	3.404 (3)	170

Symmetry codes: (i) *x*−1/2, −*y*+1/2, *z*−1/2.
